# Remote sensing of alcohol consumption using machine learning speckle pattern analysis

**DOI:** 10.1117/1.JBO.30.3.037001

**Published:** 2025-03-04

**Authors:** Doron Duadi, Avraham Yosovich, Marianna Beiderman, Sergey Agdarov, Nisan Ozana, Yevgeny Beiderman, Zeev Zalevsky

**Affiliations:** a Bar Ilan University, Faculty of Engineering and Nanotechnology Center, Ramat Gan, Israel; b Ruppin Academic Center, Faculty of Engineering, Kfar Monash, Israel; c Holon Institute of Technology, Faculty of Electrical and Electronic Engineering, Holon, Israel

**Keywords:** speckle, alcohol, remote sensing, machine learning

## Abstract

**Significance:**

Alcohol consumption monitoring is essential for forensic and healthcare applications. While breath and blood alcohol concentration sensors are currently the most common methods, there is a growing need for faster, non-invasive, and more efficient assessment techniques. The rationale for our binary classification relates to law enforcement applications in countries with strict limits on alcohol consumption such as China, which seeks to prevent driving with even the smallest amount of alcohol in the bloodstream.

**Aim:**

We propose a remote optical technique for assessing alcohol consumption using speckle pattern analysis, enhanced by machine learning for binary classification. This method offers remote and fast alcohol consumption evaluation without requiring before and after comparisons.

**Approach:**

Our experimental setup includes a laser directed toward the subject’s radial artery, a camera capturing defocused speckle pattern images of the illuminated area, and a computer. Participants consumed alcohol and were tested periodically. We developed a machine learning classification model that performs automatic feature selection based on temporal analysis of the speckle patterns. The model was evaluated using various labeling schemes: classification with five labels, consolidation to three labels by merging similar labels, and three different binary classifications cases (“Alcohol” or “No alcohol”).

**Results:**

Our classification models showed improving accuracy as we reduced the number of labels. The initial five-label model achieved 61% accuracy. When consolidated into three labels, the models achieved accuracies of 74% and 85% for the two cases. The binary classification models performed best, with model A achieving 91% accuracy and 97% specificity, model B achieving 83% accuracy, and model C achieving 88% accuracy with 99% sensitivity.

**Conclusions:**

Our binary classification model C can successfully distinguish between pre- and post-alcohol consumption with high sensitivity and accuracy. This performance is particularly valuable for clinical and forensic applications, where minimizing false negatives is crucial.

## 1 Introduction

Alcohol consumption is a prevalent practice worldwide, deeply ingrained in various social, cultural, and individual contexts. Its impact on health and society is intricate and varied. Regarding health, alcohol’s effects range from beneficial to harmful, depending on the amount consumed. Alcohol consumption affects physiological properties such as heart function, the skin’s antioxidant network, and its optical properties.^1^ Although alcohol consumption can have both positive and negative health outcomes,^2^^,^^3^ the absence of a universally agreed-upon definition for moderate drinking underscores its subjective nature. These biological consequences transcend individual health concerns to precipitate social problems, such as accidents resulting in traffic fatalities. Driving under the influence of alcohol ranks among the leading causes of car accidents and fatalities worldwide.^4^ Even at blood alcohol concentrations below the legal limit, alcohol can significantly compromise driving abilities.^5^ Consequently, the assessment of alcohol-induced intoxication or consumption is crucial from both forensic and pharmacological perspectives.^6^ Efforts to mitigate the dangers of alcohol-impaired driving involve a multifaceted approach, including legislative measures, law enforcement, public awareness campaigns, and technological advancements.^7^^,^^8^ Law enforcement agencies conduct sobriety checkpoints and employ tests to identify and apprehend individuals driving under the alcohol influence.^9^

The primary method utilized by law enforcement for testing alcohol levels is the breathalyzer device, which measures breath alcohol concentration/content (BrAC). The BrAC examination is based on the premise that it is possible to measure or approximate the alcohol concentration in the alveolar air space. This test relies on the rapid equilibration of alcohol, occurring within 2 ms, as it passes between pulmonary capillary blood and alveolar air. During this process, alcohol molecules diffuse into both the airways and various body tissues. The BrAC test is fast, non-invasive, and straightforward and provides immediate results, facilitating the prompt charging of suspects. However, it requires the subject’s cooperation, which can be challenging in situations involving combative or trauma-afflicted individuals. Moreover, the BrAC test is susceptible to various factors, including the potential for false positives due to molecules resembling ethanol, genetic predisposition,^10^^,^^11^ variations in body temperature, breath compounds, breath-to-blood ratio variations, device calibration, and human error during test administration. For alcohol levels around 0.05%, studies have reported that standard breathalyzers achieve a sensitivity of 93.2% and specificity of 96.1%.^12^ Another study, evaluating breathalyzer performance at higher BACs (≥0.05%), reported sensitivity and specificity values of 97.2% and 100%, respectively.^13^ These findings reinforce breathalyzers’ reliability in detecting high BACs, particularly for intoxication thresholds. However, such measurements require direct contact and individual testing, which limits their effectiveness in large-scale and rapid monitoring scenarios. These limitations emphasize the importance of considering contextual factors and corroborating evidence when using breathalyzers results in legal proceedings.^14^

A more precise assessment of alcohol consumption involves determining blood alcohol concentration/content (BAC). BAC offers a direct measurement of the amount of alcohol present in the bloodstream. The process of measuring BAC involves a rapid dispersion of orally consumed alcohol, which circulates through the bloodstream and reaches various organs, including the brain, liver, and kidneys.^15^ Once in the bloodstream, alcohol undergoes metabolism in the liver through enzymes at a specified decomposition rate.^16^^,^^17^ Consequently, the concentration decreases linearly over the hours following alcohol consumption. The existing BAC calculator allows estimating blood alcohol levels based on factors such as the amount of alcohol consumed, gender,^18^ body weight,^19^^,^^20^ alcohol strength, and drinking duration. However, it should be used as a guideline rather than a definitive measure, as individual variations in metabolism, tolerance, hydration levels, and other factors can affect alcohol absorption and elimination rates. BAC limits for driving vary globally, ranging from 0% to 0.08%. The vast majority of countries set the limit at 0.05%, whereas some have stricter limits, such as 0.02% or even 0%. Some countries impose a BAC limit of 0.01% for young drivers as lower BAC laws have been shown to reduce alcohol-related crashes. Therefore, it is essential to exercise caution and discretion when interpreting BAC calculator results and always prioritize safety when consuming alcohol.^21^

To address the demand for a faster and more efficient evaluation of BAC, the development of new measurement methods is imperative. These methods should prioritize accuracy, reliability, high sensitivity, rapid response time, cost-effectiveness per test, portability, and noninvasive procedures. Various noninvasive techniques have been developed to assess blood alcohol content.^22^ For example, biosensing devices can monitor alcohol levels in urine,^23^ sweat,^24^ and saliva.^25^ Other alternative methods include iodometric titrations, electrochemical ethanol measurements, and noninvasive optical techniques. Noninvasive optical techniques encompass various approaches, such as refractive index measurement for detecting alcohol in liquid solutions,^26^ diffuse reflectance near-infrared spectroscopy for *in vivo* tissue alcohol testing,^27^^,^^28^ and horizontal attenuated total reflectance-Fourier transform infrared spectrometers.^29^ By leveraging these innovative measurement techniques, it becomes possible to address the need for rapid and accurate assessment of BAC in various settings, including law enforcement, healthcare, and personal use scenarios. In addition, the portability and noninvasive nature of these methods make them particularly valuable for on-the-go or point-of-care applications.

Our optical sensor presents a noninvasive approach for remote monitoring of various biomedical parameters through the temporal analysis of speckle patterns reflected from a tissue illuminated by a laser beam. Each speckle pattern represents a reference point of a self-interfered pattern, enabling tracking of the light phase variations caused by the illuminated surface roughness.^30^ Our remote optical sensing method utilizes a laser and fast-imaging camera to observe the reflected speckle patterns while defocusing the illuminated object. When an object undergoes tiny movements, it causes a lateral shift in the speckle pattern, which can be measured through the spatial pattern correlation. The optical imaging system tracks the temporal trajectories of the speckle patterns, and a MATLAB algorithm extracts the movement from the correlation between the recorded frames. This approach has demonstrated the ability to measure various biomedical parameters,^31^ including monitoring blood pulse pressure,^32^ heart rate,^33^ blood glucose concentration,^34^ pigmented lesion,^35^ vocal fold vibrations,^36^ and cilia motion.^37^ By leveraging this optical sensing technology, medical professionals can remotely and noninvasively monitor critical physiological parameters, providing valuable insights into a patient’s health status without the need for direct contact or invasive procedures.

**Fig. 1:**
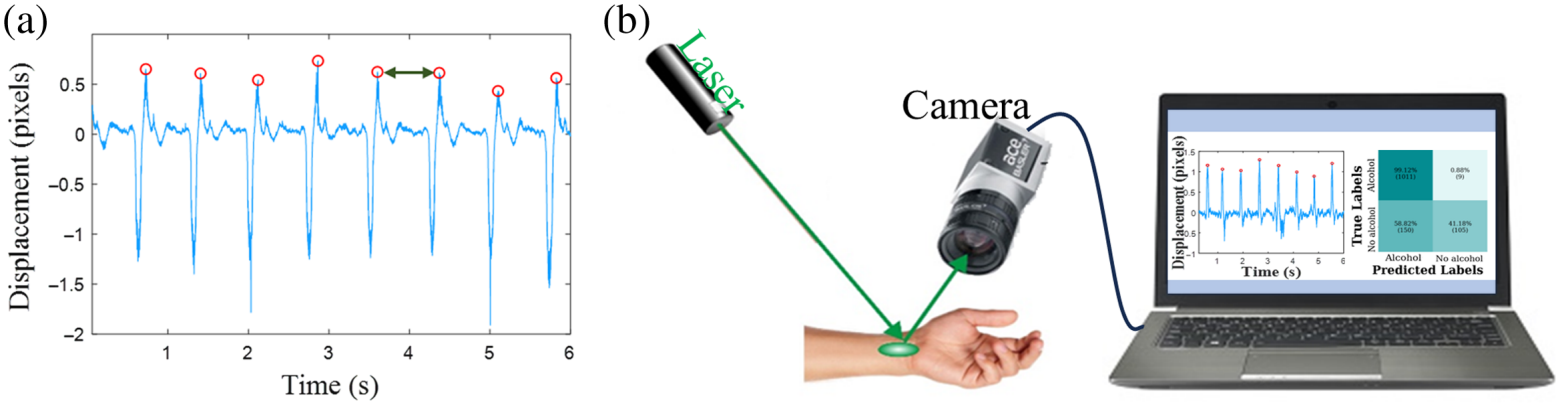
(a) Illustration of the PCG signal and (b) the optical setup for alcohol consumption sensing.

In our previous research,^38^ we introduced a remote technique for estimating alcohol concentration in the bloodstream based on analyzing speckle patterns. Blood alcohol levels influence blood viscosity and heart function, which can be detected through speckle pattern analysis. When alcohol enters the bloodstream, it affects multiple aspects of heart function, including its electrical activity, rate and shape of the heartbeat profile, structural properties, and arterial behavior. These changes are reflected in modified speckle patterns during monitoring as the speckle variations measure the phonocardiogram (PCG), which records all sounds produced during a cardiac cycle. The speckle pattern analysis enables the measurement of alcohol-induced changes in heart activity through variations in the PCG signal. An illustration of a PCG signal [Fig. 1(a)], with markings highlighting the heartbeats (peaks marked with red circles) and heart rate variability (time differences between consecutive heartbeat peaks indicated by green arrow), demonstrates how changes in heart function can be used to monitor the physiological response to alcohol consumption.

This optical technique offers several advantages over contemporary approaches. It is simpler, more precise, more accessible, and distance invariant. Being a noncontact and noninvasive optical method, it eliminates biohazard risks associated with sample handling as it does not rely on bodily fluids such as blood, sweat, or urine.

Our current research builds upon this foundation by advancing the development of a remote method for detecting alcohol consumption. This method relies on analyzing speckle patterns and involves a new step, incorporating data processing through machine learning analysis. The processed speckle pattern data are utilized to identify alcohol consumption profiles by comparing quantitative parameters before and after consumption. Using machine learning analysis enhances the precision of blood alcohol sensing compared to the displacement graph evaluation and eliminates the need for comparing measurements of an individual’s state before and after alcohol consumption, as the system automatically classifies the measurements. This approach validated with high accuracy and sensitivity allows for real-time distant monitoring of alcohol consumption, leading to a potential application for remote monitoring of alcohol intoxication, especially for law enforcement or medically supervised patients.

## 2 Methods

### 2.1 Experimental Setup

Our system [Fig. 1(b)] consists of a 532 nm wavelength laser with an output power of 0.4 mW, a Basler camera (acA1440-220 um) equipped with a 12 mm focal length lens, and a computer. The laser was directed toward the subject’s hand radial artery to capture defocused speckle pattern images of the illuminated area. The camera was positioned ∼25  cm from the tested area to capture the reflected speckle patterns at a rate of 500 frames per second (fps) for 6 s. Thus, one recording contains 3000 frames, with a temporal resolution of 2 ms. Note that the physiological signals we measure (heartbeat and changes in heart function) occur in high-frequency ranges of 0.5 to 3 Hz, which are distinct from motion artifacts that occur at frequencies lower than 0.5 Hz as has been experimentally proven previously.^39^

We conducted measurements on five subjects aged between 23 and 65 years (Table 1), following relevant guidelines and regulations. The participants were briefed about the research objectives and potential implications and provided informed consent for their participation. All subjects were healthy, were of legal drinking age, had average body weights, and had moderate alcohol consumption habits. This group included three males and two females. During the experiment, the subjects consumed 40% vodka in doses tailored to achieve a critical blood alcohol concentration as determined by a BAC calculator, considering factors such as gender, weight, and age of the participants. Initially, the average BAC of the participants immediately after alcohol consumption was 0.05%, and it decreased linearly to an average of 0.025% over the testing period. Each subject underwent five sets of periodic measurements using our optical system and a reference alcohol breath-analyzer: before consuming alcohol, immediately after drinking, and at three additional 30-min intervals. Each recorded set comprised five speckle pattern videos, resulting in a total of 125 videos per subject.

**Table 1:** Characteristics of the tested participants.

Participant	Gender	Age (years)	Body weight (kg)	Amount of alcohol consumed (mL)
1	Female	23	51	50
2	Male	26	72	75
3	Female	28	58	60
4	Male	34	68	75
5	Male	65	70	75

### 2.2 Speckle Pattern Analysis

The analysis was conducted by tracking the temporal changes in back-scattered speckle patterns, generated by illuminating the area of interest with a laser beam.^40^ As previously mentioned, alterations in blood alcohol levels influence blood vessel and heart behavior, which is reflected in speckle pattern displacement.

To capture the speckle patterns,^41^ the optics were configured to defocus the inspected area, allowing the camera to focus on the far field. Under these conditions, the tilting movement of the illuminated surface causes linear shifts in the speckle patterns rather than changes in the patterns themselves. This tilting movement results in space-dependent phase changes, which translate into lateral shifts in the speckle patterns along the transversal plane (x and y axes). These lateral shifts are expressed through the Fourier transform as a displacement of the speckle patterns, enabling measurement of the object’s 2D displacement by tracking the temporal changes in correlation peak intensity.^42^ The processing of captured images involved calculating the correlation between two consecutive frames of the time-varying speckle patterns. By identifying the location of the maximum correlation peak between each pair of frames, the temporal change in the peak’s position was extracted. Analyzing the temporal dependency of the correlation peak’s position indicates alcohol-induced physiological variations.

**Fig. 2:**
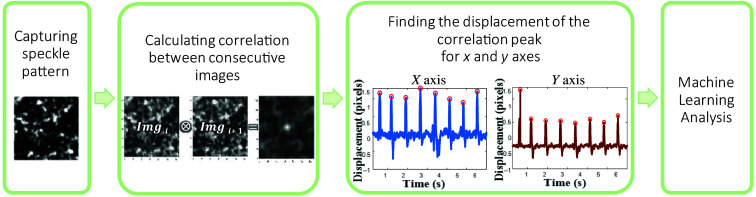
Workflow of the speckle pattern image processing for alcohol consumption estimation.

This workflow (Fig. 2) demonstrates the system’s ability to capture and process information efficiently, providing valuable data for alcohol estimation within a reasonable timeframe. Previously, displacement measurements were analyzed by evaluation of amplitude and frequency. The proposed method uses this displacement measurement as the input for machine learning, enabling the detection of additional features and improving prediction accuracy.

### 2.3 Speckle Pattern Machine Learning

#### 2.3.1 Data preprocessing

First, we divided each video into frame chunks with a length of 256 frames and a stride of 32, using overlapping to increase the number of chunks for model training. Then, we extracted four elements from the analysis of these frame chunks: displacement of the correlation peak in the X and Y axes, and change in the displacement in the X and Y axes. The measurements were taken at five time points, each with a specific label: “before drinking” (before consuming alcohol), “0 min” (immediately after drinking), “30 min” (half an hour after drinking), “60 min” (one hour after drinking), and “90 min” (one and a half hours after drinking). We divided the data from the five subjects into 80% for training and 20% for validation. This data splitting is crucial as it allows us to train the model on one subset of the data (four subjects) while evaluating its performance on another subset (one subject), ensuring that the model can generalize effectively to new, unseen data.

#### 2.3.2 Model configuration

A comprehensive set of features was extracted from each frame chunk using the “tsfresh” Python package.^43^ This package is particularly adept at automatically calculating a wide range of time-series features, crucial for capturing the intricate details and variations in speckle patterns across the frames. Utilizing “tsfresh,” we extracted features for each of the four elements, including statistical characteristics (mean, median, standard deviation) and more complex descriptors (skewness, kurtosis, quantile values). These features provide deeper insights into the underlying data dynamics.

Following feature extraction, we applied a feature selection process to the training elements to retain the most predictive features for the classification task while discarding uninformative ones. This selection process employed the “tsfresh” feature filtering method, which uses hypothesis tests to calculate the significance of features with respect to the target labels. Features with a p-value greater than 0.05 were discarded, ensuring that the model was trained on the most relevant and influential features.^44^ This approach enhances model accuracy by eliminating uninformative features, thereby reducing training time and mitigating overfitting risks associated with irrelevant feature correlations.

Subsequently, we conducted model training using XGBoost, an ensemble machine learning algorithm based on decision trees, renowned for its effectiveness in classification tasks, particularly with large, complex datasets and high-dimensional feature spaces. We then performed hyperparameter tuning using the Optuna framework, a robust method for automatically identifying optimal parameters. This optimization focused on key parameters of the XGBoost model, including the number of trees, tree depth, and learning rate, optimizing each tree’s contribution to final predictions. Optuna systematically tested various parameter combinations to maximize accuracy on the validation set. Once optimal parameters were determined, we trained XGBoost on the entire training dataset. This iterative training process involved constructing trees and adjusting the model to minimize errors, thereby improving predictive accuracy.

**Fig. 3:**
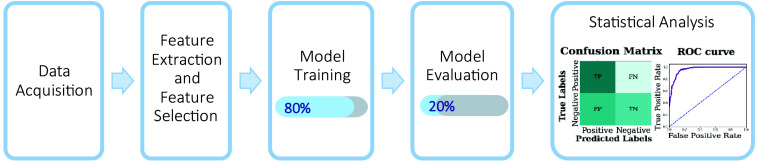
Workflow of the machine learning model for alcohol consumption estimation.

This workflow (Fig. 3) allows determining an appropriate number of labels required for the classification model based on our results. We comprehensively evaluated the trained model’s performance on the validation set using metrics such as accuracy, precision, and sensitivity to ensure it met the expected standards of reliability and effectiveness in classifying different time measurements of alcohol consumption.

The classification results are presented in a confusion matrix, with its size varying depending on the number of labels in the classification model. Each cell in the matrix shows the accuracy percentage per label and, in parentheses, the number of measurements. For binary classification, the confusion matrix is divided into four cells, with “alcohol” as the positive label. The cells represent: true positive (TP), where both true and predicted labels are “alcohol”; false negative (FN), where the true label is “alcohol” but predicted as “no alcohol”; true negative (TN), where both true and predicted labels are “no alcohol”; and false positive (FP), where the true label is “no alcohol” but predicted as “alcohol.” For each case, we calculated the following metrics: accuracy [Eq. (1)], precision [Eq. (2)], sensitivity [Eq. (3)], specificity [Eq. (4)], and F1 score [Eq. (5)].

$$\text{Accuracy}=\frac{\mathrm{TP}+\mathrm{TN}}{\mathrm{TP}+\mathrm{FP}+\mathrm{TN}+\mathrm{FN}},$$ (1)

$$\text{Precision}=\frac{\mathrm{TP}}{\mathrm{TP}+\mathrm{FP}},$$ (2)

$$\text{Sensitivity}=\frac{\mathrm{TP}}{\mathrm{TP}+\mathrm{FN}},$$ (3)

$$\text{Specificity}=\frac{\mathrm{TN}}{\mathrm{TN}+\mathrm{FP}},$$ (4)

$$F1\text{ score}=\frac{2\cdot \text{Precision}\cdot \text{Sensitivity}}{\text{Precision}+\text{Sensitivity}}.$$ (5)

The F1 score is commonly used to evaluate the predictive performance of the classification models. F1 is calculated from precision and sensitivity; thus, high precision and sensitivity result in a high F1 score, indicating a reliable model. In addition, we obtained the receiver operating characteristic (ROC) curve, which shows the performance of the classification model at all classification thresholds. The area under the ROC curve (AUC) is used as a measure of the overall performance.^45^

## 3 Results and Discussion

**Fig. 4:**
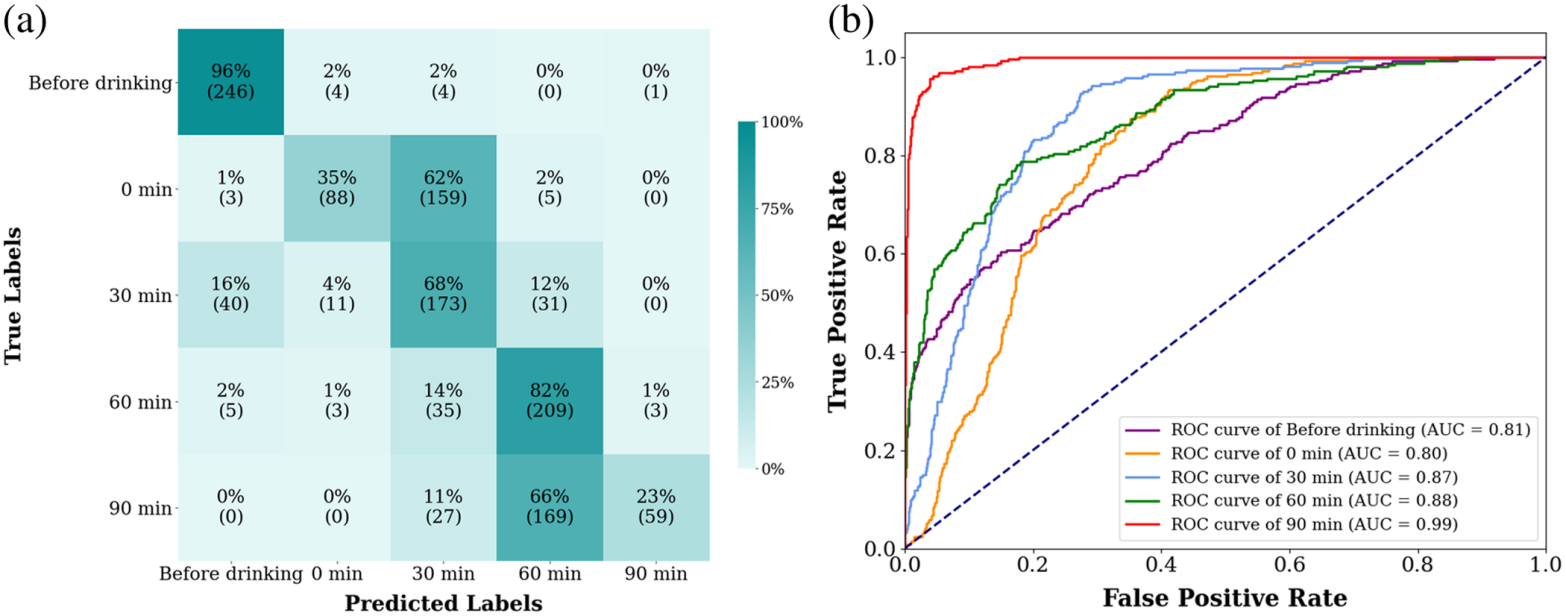
Model of five labels. (a) Confusion matrix and (b) ROCs.

First, we processed the measurements classified into five labels using the developed classification model. The confusion matrix [Fig. 4(a)] allows distinguishing between the labels “before drinking” and “60 min.” Specifically, the label “before drinking” has a high accuracy of 96%. However, the label “0 min” shows a lower accuracy of 35%, indicating uncertainty in its distinction as the model often classifies it as “30 min.” The accuracy for the label “30 min” is 68%, with occasional misclassifications as “before drinking,” “60 min,” or “0 min.” The label “60 min” has a high accuracy of 82% but is sometimes misclassified as “30 min.” In addition, the label “90 min” has a low accuracy of 23%, meaning the distinction is uncertain, as the model primarily classified it as “60 min” and occasionally as “30 min.” The overall model accuracy is 61%. We observed from the ROC curves [Fig. 4(b)] that the labels had good AUC values (AUC≥0.8), and the label “90 min” had the highest AUC of 0.99.

**Fig. 5:**
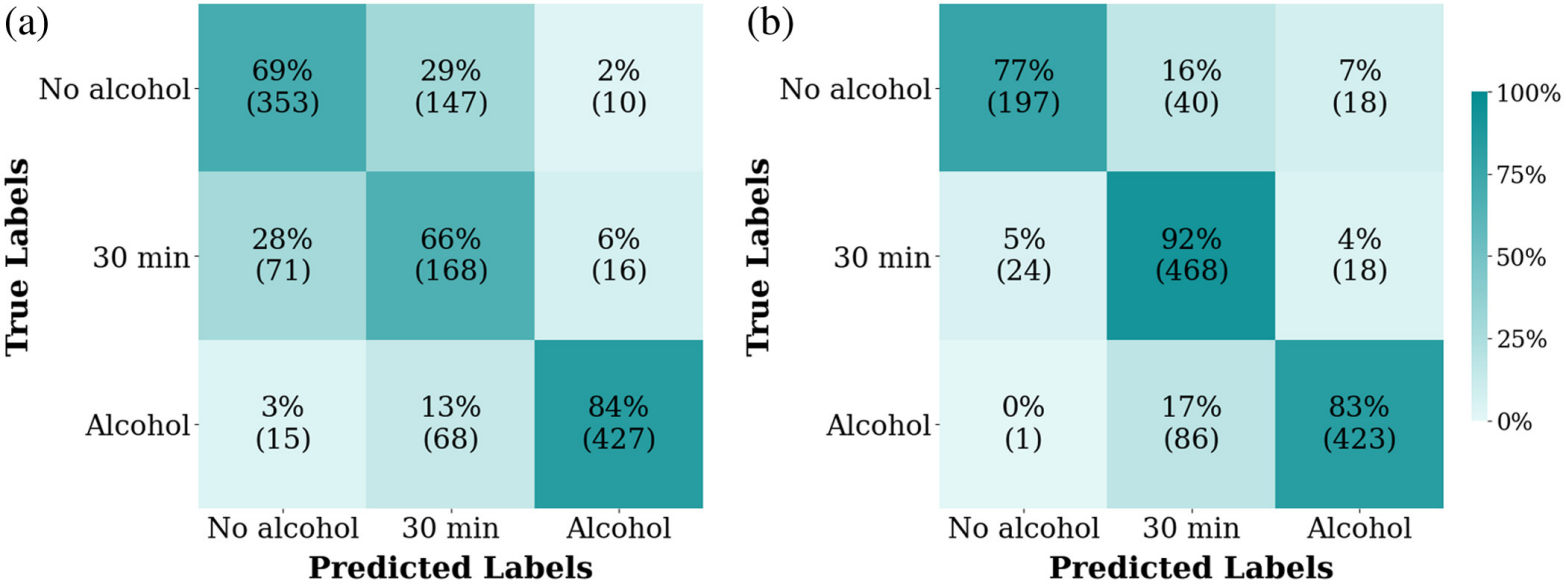
Model of three labels. Confusion matrix (a) for the first case and (b) for the second case.

Based on these results, we decided to merge some labels and retrain the model. We divided the model into three labels for two cases. In the first case, “before drinking” and “0 min” are labeled as “no alcohol;” “60 min” and “90 min” are labeled as “alcohol;” and the last is labeled as “30 min.” The confusion matrix [Fig. 5(a)] shows that the label “alcohol” has a high accuracy of 84% but is sometimes classified as “30 min.” The label “no alcohol” has an accuracy of 69% but is sometimes classified as “30 min.” The label “30 min” has an accuracy of 66% but is sometimes classified as “no alcohol.” The overall model accuracy for this case is 74%. In the second case, “before drinking” is labeled as “no alcohol;” “60 min” and “90 min” are labeled as “alcohol;” and the last is labeled as “0 and 30 min.” From the confusion matrix [Fig. 5(b)], we observed that the label “alcohol” has a high accuracy of 83% but is sometimes classified as “0 and 30 min.” The label “no alcohol” has an accuracy of 77% but is sometimes classified as “0 and 30 min.” The label “0 and 30 min” has a high accuracy of 92%. The overall model accuracy for this case is 85%.

**Fig. 6:**
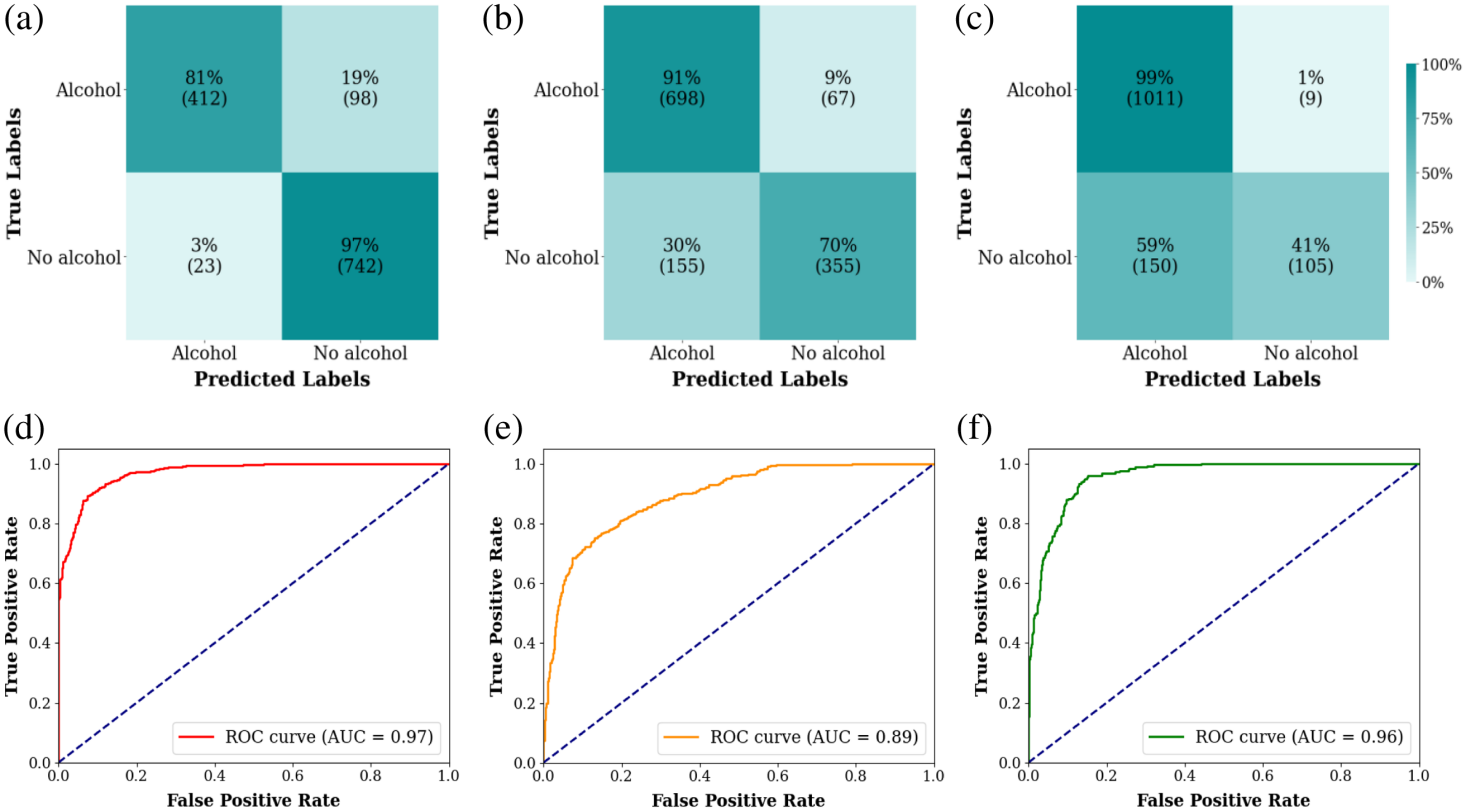
Models of two labels. Confusion matrix; (a) model A, (b) model B, (c) model C, and ROC curves; (d) model A, (e) model B, (f) model C.

Considering the obtained results, we decided to switch to binary classification and retrained the model. We classified the measurements into three cases, each with only two labels: “no alcohol” and “alcohol.” In model A, “before drinking,” “0 min,” and “30 min” are labeled as “no alcohol,” whereas “60 min” and “90 min” are labeled as “alcohol.” In model B, “before drinking” and “0 min” are labeled as “no alcohol,” whereas “30 min,” “60 min,” and “90 min” are labeled as “alcohol.” In model C, “before drinking” is labeled as “no alcohol,” and “0 min,” “30 min,” “60 min,” and “90 min” are labeled as “alcohol.” The confusion matrices [Figs. 6(a)–6(c)] show that the accuracies of models A, B, and C are 91%, 83%, and 88%, respectively. The ROC curves [Figs. 6(d)–6(f)] show that models A, B, and C have high AUCs of 0.97, 0.89, and 0.96, respectively. In addition, we calculated the parameters from Sec. 2.3 and presented them in Table 2 for comparison between the three cases of the model.

**Table 2:** Binary classification model results for the three models.

Model	Accuracy (%)	Precision (%)	Sensitivity (%)	Specificity (%)	F1 score (%)
A	91	95	81	97	87
B	83	82	91	70	86
C	88	87	99	41	93

## 4 Conclusions

The proposed method presents a remote and real-time optical approach for estimating alcohol consumption, based on a machine-learning algorithm applied to classify speckle patterns reflected from skin. Initially, we classified the measurements into five labels based on consecutive measurements. However, distinguishing between certain labels proved uncertain. Consequently, we merged labels into two distinct cases to evaluate the model’s recognition performance, reducing the classification to three labels. This process aided in selecting appropriate label-merging strategies. We then presented three distinct cases of binary alcohol consumption classification.

Our primary goal was to identify subjects who had consumed alcohol, emphasizing the critical importance of the model’s high sensitivity to minimize the miss rate (type II error), which refers to cases where subjects who had consumed alcohol were not detected. In addition, achieving high specificity is essential to reduce the probability of false alarms (type I error), which refers to cases where sober subjects were incorrectly identified as having consumed alcohol. Given the trade-off between sensitivity and specificity, we prioritized high sensitivity. This prioritization aligns with our objective of developing a remote, real-time optical screening tool for detecting alcohol consumption, where identifying all potential cases takes precedence over reducing false alarms. Although lower specificity may lead to more false alarms, these can be effectively addressed through secondary confirmation methods, such as portable breathalyzers, to validate initial detections. This approach is particularly valuable in real-world scenarios where any alcohol consumption is prohibited, such as traffic safety, workplace environments, or public events.

In our analysis of binary classification models, model C showed high sensitivity (99%) but low specificity (41%). By contrast, model A presented high specificity (97%) but lower sensitivity (81%). Model A achieved the highest precision (95%), while model C achieved the highest F1 score (93%). Considering accuracy, specificity, and precision, model A performed the best overall. However, prioritizing sensitivity over specificity led us to prefer model C despite its lower specificity. Model C’s accuracy, AUC, and precision were comparable to those of model A, with significantly higher sensitivity, making it our preferred choice.

Model C’s binary classification distinguishes between pre-alcohol consumption (labeled as “before drinking”) and all post-alcohol consumption measurements (labeled as “0 min,” “30 min,” “60 min,” and “90 min”). Its ability to differentiate between pre-consumption and post-consumption with high sensitivity is highly satisfactory, given the importance of accurately identifying subjects who have consumed alcohol with certainty. This capability is crucial because many countries restrict driving under any blood alcohol concentration, creating a need for a fast, remote, and accurate identification of alcohol consumption.

However, it is essential to note that the accuracy and reliability of such models depend on factors such as the quality of speckle pattern data, the robustness of feature extraction algorithms, and the diversity and representativeness of the training dataset. These aspects significantly influence the model’s performance and effectiveness in alcohol consumption estimation.

Note that the system’s accuracy was evaluated using a relatively small dataset. To enhance the robustness of our analysis, we implemented a data augmentation technique to artificially expand our dataset size, which significantly increased our sample size. The data augmentation helped prevent overfitting and improved model generalization by creating modified versions of existing data, making our model more robust without requiring additional data collection. Although testing was performed on a single subject, the temporal nature of our data (multiple measurements across different time points) allowed us to evaluate the model’s ability to generalize across different blood alcohol concentrations and time periods.

Our next step is to conduct experiments involving a large, diverse group of subjects spanning various ages (young and old), genders, weights, and different alcohol consumption levels. This approach aims to develop a sophisticated and comprehensive model for accurately characterizing alcohol consumption.

## Data Availability

The data that support the findings of this article are not publicly available due to subject privacy concerns but are available from the corresponding author upon reasonable request.
